# Pro- and Anti-Inflammatory Cytokines’ Implication in Right Heart Disease, Arrhythmogenesis, and Atrial Fibrillation

**DOI:** 10.3390/biom16020216

**Published:** 2026-02-01

**Authors:** Danyl Khider, Orlane Neuilly, Roddy Hiram

**Affiliations:** 1 Department of Medicine, Faculty of Medicine, University of Montreal, Montreal, QC H3T 1J4, Canada; 2 Montreal Heart Institute, Montreal, QC H1T 1C8, Canada

**Keywords:** inflammation, fibrosis, cardiac arrhythmias, atrial fibrillation, right heart disease

## Abstract

Atrial fibrillation (AF) is the most common cardiac arrhythmia responsible for increased risk of a stroke and sudden death. Right heart disease (RHD), characterized by myocardial dysfunction and structural alteration affecting the right ventricle and the right atrium, is recognized as an important risk factor for AF. Inflammation and cardiac fibrosis emerge as arrhythmogenic pathophysiological events commonly occurring in most diseases responsible for cardiac arrhythmias and AF. However, no commercially available anti-inflammatory drugs were shown to irreversibly cure AF. Hence, more investigations are required to identify the key inflammatory and fibrosis agents involved in arrhythmogenesis. In this review, we explore (i) the recent evidence of myocardial, and especially atrial, inflammation in RHD, and (ii) the relevance of targeting inflammatory and anti-inflammatory cytokines in future strategies, combined with current AF management, in RHD.

## 1. Introduction

Atrial fibrillation (AF) is the most common sustained arrhythmia encountered in clinical cardiology and represents a major contributor to morbidity, strokes, and heart failure [1]. AF arises from a complex interplay between structural, electrical, and metabolic remodeling of the atrial myocardium that disrupts the normal synchronized propagation of electrical impulses [2]. Although AF has classically often been associated with left-sided cardiac diseases such as hypertension, pulmonary vein ectopy, or mitral valve dysfunction, recent evidence highlights its increasing prevalence in right heart disease (RHD), particularly under conditions of pulmonary hypertension (PH) or chronic right ventricular overload [3,4,5].

**Figure 1:**
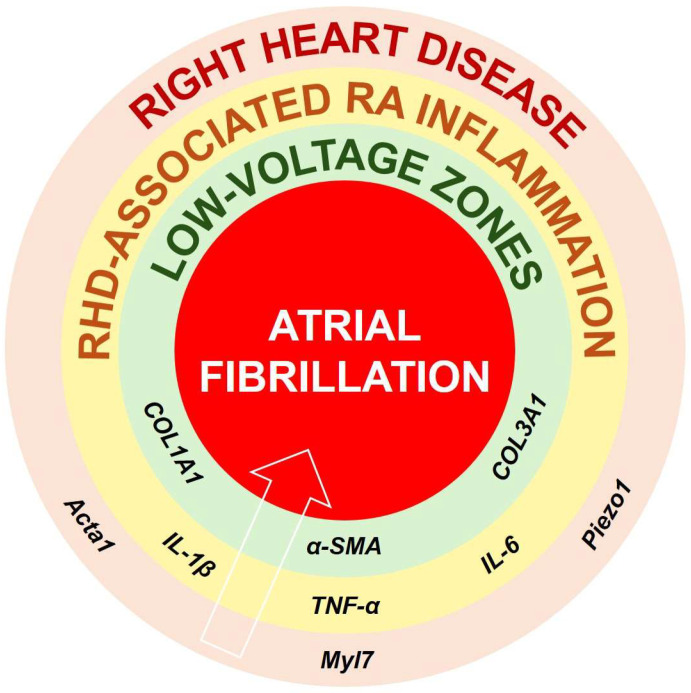
Proposed pathophysiological connections between RHD, specific inflammatory cytokines, structural and electrical remodeling, and the onset or maintenance of AF. It is suggested that RHD is accompanied by expression of markers of hypertrophy and dilation such as *Acta1*, *Myl7*, or *Piezo1*. Such remodeling might initiate RV and RA inflammation involving IL-1β, IL6, and TNF-α, which, if not attenuated, can stimulate the production and maintenance of COL1A1, COL3A1, or α-SMA. Persistent and irreversible RA fibrosis deposits can be responsible for formation of low-voltage zones, perturbed conduction and arrhythmogenesis, and AF. Abbreviations: α-SMA, alpha smooth-muscle actin; Acta1, alpha actin 1; COL, collagen; IL, interleukin; Myl7, myosin light chain 7; Piezo1, piezo-type mechanosensitive ion channel component 1; TNF-α, tumor necrosis factor alpha.

RHD refers to a group of conditions in which the right ventricle (RV) and the right atrium (RA) become hypertrophic, dilated, and eventually fail (in case of right heat failure [RHF]), as a result of chronic pressure or volume overload [3,4] (Figure 1). Studies have identified that some markers of hypertrophy, such as *Myl7*, *Piezo1*, and *Acta1*, contribute to myocardial hypertrophy and dilation in RHD [3,6]. Pulmonary vascular remodeling, chronic hypoxia, and tricuspid regurgitation are frequent triggers of RHD [3,5,6]. As the RV and RA adapt to persistent overload, a reduced oxygen supply and increased oxidative stress cause myocardial injury, which promotes the recruitment of inflammatory cells and activates immune signaling pathways [5,7]. The ensuing release of pro-inflammatory mediators, including interleukin-(IL)-6, tumor necrosis factor-α (TNF-α), or IL-1β, contributes to both local and systemic inflammation [5,7,8].

Hence, inflammation is increasingly recognized as a potential key driver of RHD progression, contributing to both structural remodeling and functional impairment of the RV and RA [8,9]. Elevated circulating levels of pro-inflammatory cytokines, such as IL-6, IL-1β, and TNF-⍺, have been consistently reported in patients with PH and RHD [10]. These mediators promote endothelial dysfunction, oxidative stress, and cardiomyocyte apoptosis, thereby exacerbating pulmonary vascular remodeling and increasing RV afterload [11] (Figure 1). At the myocardial level, TNF-⍺, IL-1β, and IL-18 might impair cardiomyocyte contractility by disputing calcium handling and mitochondrial energetics, while also stimulating fibroblast proliferation, leading to fibrosis [12]. IL-6 may contribute to maladaptive hypertrophy through activation of the inflammasome NLRP3 (NOD-like receptor family pyrin domain containing 3) signaling pathway, which could promote metabolic inefficiency and contractile dysfunction in chronic overload states [5,13] (Figure 1).

Conversely, anti-inflammatory mediators such as IL-10 and resolvin (Rv)-D1 have been shown to counterbalance these processes by suppressing nuclear factor kappa B (NF-kB) activation, reducing oxidative stress, and improving RV functional adaptation under pressure overload [14,15]. An imbalance between pro- and anti-inflammatory signaling pathways has emerged as a central mechanism linking inflammation to the pathogenesis of RHD [9,15] (Figure 1). It has been suggested that the inflammatory response to RHD-induced myocardial remodeling can lead to chronic pro-fibrosis signals and irreversible fibrosis development, characterized by RA and RV expression of collagen type I and III (COL1A1, COL3A1) [9,10] (Figure 1). Such development of RA fibrosis might be responsible for arrhythmogenesis and AF (Figure 1).

Therapeutic modulation of cytokine networks could therefore represent a promising adjunct to conventional afterload-targeting strategies in preventing right heart decompensation [16].

In this narrative review paper, we aim to discuss (i) the role of pro-inflammatory cytokines in the progression of AF and RHD; (ii) the action of anti-inflammatory cytokines in the prevention of AF and RHD; and finally, (iii) the relevance of targeting inflammatory biomarkers in the management of AF and RHD.

Overall, although mounting evidence suggests a role of inflammation in AF, few data are available about its implication in RHD. Hence, the novelty of this narrative review article relies on the understanding of the pathophysiology of AF, focalizing on the specific context of myocardial inflammation in RHD.

## 2. Methods for Literature Search

Clinical and basic research reports and recent review articles were identified as related to AF and RHD in PubMed, MEDLINE, Science Direct, and Scopus, from their inception until January 2026.

The following search formula was used: (“right heart disease” OR “right heart failure” OR “right-sided cardiac disease”) [title/abstract] (“heart”, “cardiac”) [title/abstract] (“atrial fibrillation”), “proinflammatory” AND “anti-inflammatory”, “cytokines” AND “interleukins”, and “biomarkers”, “agents” AND “fibrosis”.

Additional articles were identified based on citations in the papers cited. Only articles with English full texts were reviewed.

## 3. Role of Pro-Inflammatory Mechanisms and Cytokines in the Progression and Maintenance of Right Heart Disease and Arrhythmias, Including Atrial Fibrillation

### 3.1. Generalities

Recent evidence suggests that inflammation might play a central role in the association between RV chronic mechanical stress, structural changes affecting the myocardium, and the development of cardiac arrhythmias, including AF, in RHD [4,17,18]. Under conditions of sustained right-sided pressure or volume overload, such as those caused by pulmonary vascular disease or tricuspid insufficiency, cardiomyocytes (CMs), fibroblasts (FBs), and endothelial cells (ECs) experience mechanical and oxidative stress that trigger immune cell recruitment and cytokine production [19]. Studies have suggested that, among the principal inflammatory mediators involved in RV remodeling, IL-1β, IL-6, and TNF-α are consistently elevated in patients and experimental models of RHD and correlate with disease severity [20,21]. It has been suggested that these cytokines are secreted by activated macrophages, ECs, FBs, and stressed CMs, creating a self-sustaining inflammatory loop that drives tissue injury and maladaptive cardiac remodeling, promoting arrhythmogenesis [22].

### 3.2. Basic Studies Demonstrating the Implication of Pro-Inflammatory Signals Associated with Arrhythmias in RHD

#### 3.2.1. Preclinical Evidence of Structural Arrhythmogenic Cardiac Remodeling Driven by Inflammatory Cytokines in RHD

Pro-inflammatory cytokines profoundly disturb the homeostasis of the extracellular matrix [23]. In a rat model of RHD provoked by a single injection of monocrotaline (MCT), chronic mechanical overload exposed the RA myocardium to sustained inflammatory signaling, as identified by elevated levels of NLRP3, ASC (apoptosis-associated speck-like protein containing PYCARD), CASP8 (caspase-8), IL-1β, TGFβ3 (transforming growth factor beta 3), CXCL1 (C-X-C motif chemokine ligand 1), or CXCL2 [5,15]. Pro-inflammatory biomarkers were shown to promote FB activation and extracellular matrix deposition [23,24]. A study performed in rats exposed to MCT and Sugen–hypoxia confirmed that progressive alteration of the RV structure in RHD might be accompanied by increased levels of pro-inflammatory agents such as TNF-⍺ and CRAR1 (Complement 5a Receptor 1) [25]. In these animal models, cytokine-driven RA fibrosis, identified by elevated levels of COL3A1 (collagen type 3a1) or a-SMA (alpha-smooth muscle actin) [5,25], might reduce the electrical continuity across the RA wall and increase regional conduction heterogeneity, which may favor the formation of re-entry circuits and cardiac arrhythmias [26,27]. In a canine model of pulmonary artery banding (PAB), persistent RV failure was accompanied by elevated levels of IL-1β and IL6 [28]. In addition, pulmonary arterial hypertension and RV failure induced in Wistar rats revealed elevated myocardial and septal levels of TNF-α, but no sign of inflammation was observed in the left ventricle (LV) [29].

Interestingly, experimental studies performed in rabbits, mice, and rats converged to describe IL6 and TNF-⍺ as potential activators of cardiac fibrosis, promoting the differentiation of myocardial fibroblasts and up-regulation of the synthesis of type I and III collagen, fibronectin, and matrix metalloproteinases (MMP-2/9), while suppressing tissue inhibitors of metalloproteinases (TIMPs) [30,31,32,33]. In RHD, the resulting imbalance might promote interstitial fibrosis and tissue anisotropy, creating conduction barriers in the RA [5,9]. Studies have shown that, in such a context of atrio-ventricular remodeling and hypertrophy, IL-6 could amplify the inflammatory response through JAK/STAT3 signaling, inducing myofibroblast differentiation and collagen accumulation [34,35]. In general, structural disorganization affecting the myocardium slows electrical impulse propagation and forms an arrhythmogenic substrate for re-entrant activity that might predispose to atrial arrhythmias, including AF [36] (Table 1).

#### 3.2.2. Preclinical Demonstration of the Implication of Arrhythmogenic Inflammatory Cytokines in Cardiac Oxidative Stress and Endothelial Dysfunction in RHD

Inflammatory cytokines have been shown to trigger reactive oxygen species (ROS) production via nicotinamide adenine dinucleotide phosphate (NADPH) oxidases and mitochondrial pathways [37]. In the context of RHD, rodent and canine models helped to observe that sustained elevation of myocardial levels of IL-1β, IL-6, or TNF-α contributed to enhance oxidative stress within the RV and RA, as identified by decreased heme oxygenase 1 levels and increased ROS expression, creating a biochemical environment that further destabilizes the myocardial structure and function [5,25,28,29,30,31,32,33,38]. This ROS burden was shown to directly impair endothelial nitric oxide (NO) signaling, and accumulating evidence suggests that excess ROS diminish NO bioavailability, leading to endothelial dysfunction, leukocyte adhesion, NLRP3 inflammasome activity, and microvascular ischemia [39]. Applied to the RA, these alterations might increase diastolic stiffness and promote RA dilation, thereby exacerbating mechanical stress and contributing to maladaptive remodeling, as observed in RHD [15] (Table 1).

Furthermore, cytokine-induced oxidative stress may provoke a cascade of deleterious and arrhythmogenic events [27]. When ROS synergize with IL-1β to activate the NLRP3 inflammasome, it has been shown that a self-amplifying inflammatory cycle and an arrhythmogenic substrate can emerge due to sustained chronic cytokine release [39,40,41,42,43].

**Figure 2:**
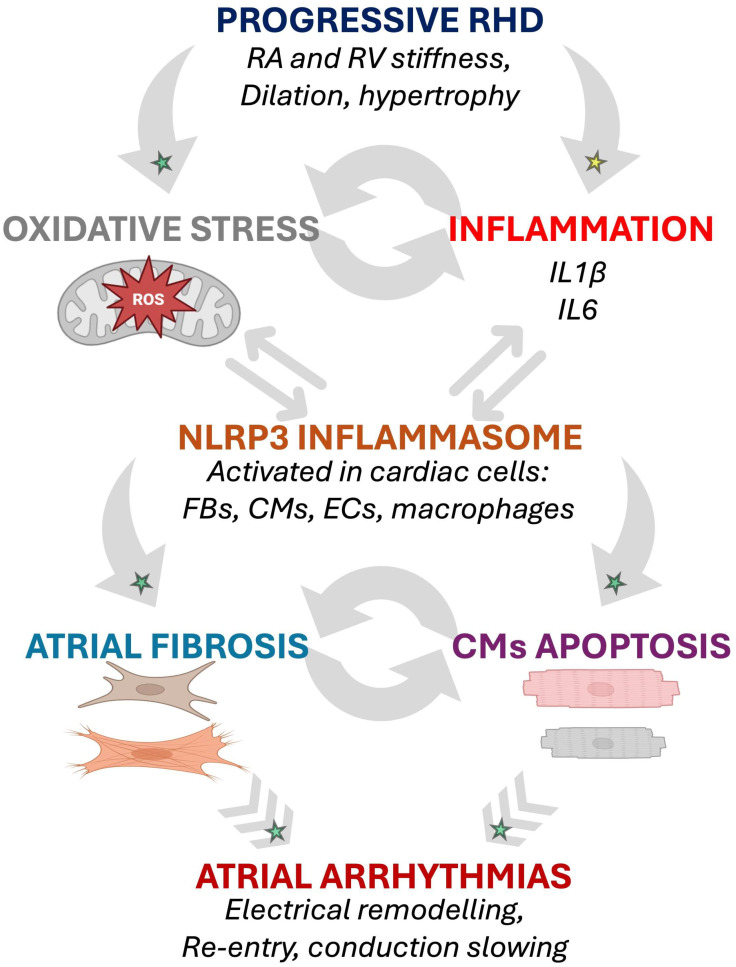
Potential inflammation- and oxidative stress-induced arrhythmogenic cellular remodeling as suggested by animal models of RHD. The progressive RA and RV myocardial remodeling observed in RHD might be associated with activation of biomarkers of oxidative stress and inflammation involved in arrhythmogenic pathophysiological changes affecting cardiac cells [15,25,28,29]. Established mechanisms in human pathophysiology are identified by a green star. Emerging/speculative pathways based on recent preclinical evidence are identified by a yellow star. Abbreviations: CM, cardiomyocyte; EC, endothelial cell; FB, fibroblast; IL, interleukin; RA, right atrium; RHD, right heart disease; ROS, reactive oxidative species; RV, right ventricle.

The pathophysiological process involving oxidative stress and inflammasome activation highlights a potential mechanism through which inflammatory cytokines contribute to electrical instability and atrial arrhythmias, including AF, in RHD (Figure 2).

#### 3.2.3. Fundamental Research Suggesting an Eventual Inflammation-Associated Electrical Remodeling and Calcium-Handling Abnormalities in RHD

Beyond the potential arrhythmogenic structural effects described above, inflammatory cytokines might also directly or indirectly alter ion channel expression and calcium (Ca^2+^) dynamics, promoting cardiac arrhythmias [44]. In rat models of RHD, it has been proposed that the progression of RHD might be accompanied by persistent exposure to inflammatory stimuli such as IL-1β, IL-6, or TNF-α, which may progressively promote the destabilization of the electrical properties of RA cardiomyocytes [5,25]. In studies performed in mice, not only focusing on the RA, it has been demonstrated that IL-1β and TNF-α might reduce the L-type Ca^2+^ current (I_CaL_) and transient outward potassium current (I_to_), affecting the action potential duration and refractory period [45]. Rat models of atrial remodeling implicating myocardial inflammation emphasized that electrophysiological changes characterized by a shortened repolarization phase and increased beat-to-beat variability could be favorable to re-entry and cardiac arrhythmias [5,46,47].

**Figure 3:**
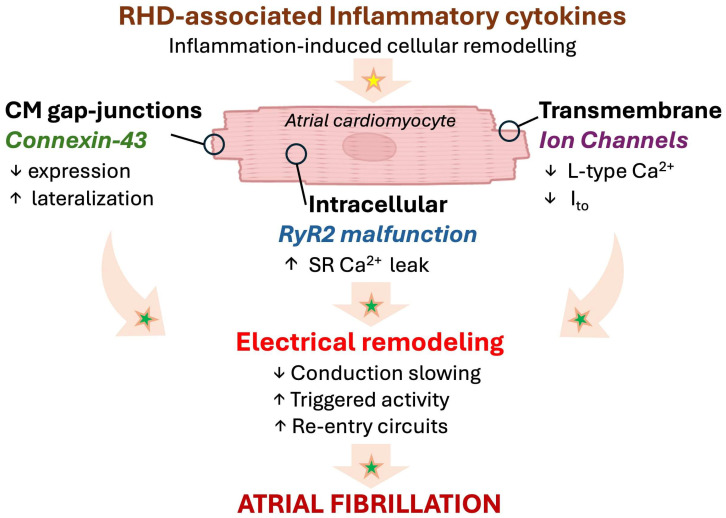
Emerging paradigm of inflammation-associated electrical remodeling with potential relevance in RHD. Progressive myocardial remodeling in RHD might provoke a local inflammatory response affecting the cardiomyocytes, leading to arrhythmogenesis and AF. Established mechanisms in human pathophysiology are identified by a green star. Emerging/speculative pathways based on recent preclinical evidence are identified by a yellow star. Abbreviations: Ca^2+^, calcium; CM, cardiomyocyte; I_to_, transient outward K^+^ channel; RHD, right heart disease; RyR, ryanodine 2 receptor; SR, sarcoplasmic reticulum.

Interestingly, in a murine model of diabetes, IL-1β has been shown to down-regulate ryanodine receptor 2, while increasing mitochondrial ROS (mitoROS), impairing conduction homogeneity, and promoting cardiac arrhythmias [48]. In the RA, reduced electrical coupling may perturb the electrical conduction pathways and promote conduction block or re-entry circuits, promoting the development of an AF substrate [5,15]. Moreover, cytokine-induced connexin 43 impairment, increased phosphorylation of ryanodine receptors, and an increased sarcoplasmic reticulum Ca^2+^ leak were described as potential triggers of delayed after-depolarizations that enhance the susceptibility for cardiac arrhythmias [49,50,51]. Together, these inflammatory and electrophysiological disturbances create a vulnerable substrate in RHD that may facilitate both the initiation and maintenance of atrial arrhythmias, including AF [5,50,51] (Figure 3).

### 3.3. Clinical Evidence of an Arrhythmogenic Inflammatory Profile in RHD

#### 3.3.1. Right Heart Inflammation Accompanied with Structural and Functional Changes in Patients with RHD

Clinical investigations increasingly demonstrate that inflammatory activation could be considered a measurable and prognostically relevant feature of RHD and RHF in humans, complementing extensive preclinical evidence. Elevated circulating pro-inflammatory cytokines have been documented in patients with pulmonary hypertension (PH) and RHD [52]. In a clinical trial involving 53 patients, serum levels of IL-1β and IL-6 were shown to be elevated in patients with primary pulmonary hypertension and COPD [52]. In a prospective study of 112 COPD patients, elevated levels of hs-CRP (high-sensitivity C-reactive protein) and IL6 were associated with severe PH [53]. In a cross-sectional study involving 97 patients with COPD, patients with PH had significantly increased circulating levels of IL6 and hs-CRP [54]. A recent multiplex protein assay performed on blood samples collected in the inferior vena cava during right heart catheterization in patients with RHF revealed increased levels of CXCL12 and soluble CD163 (cluster of differentiation 163) [55]. In a study on 40 patients with PAH, the investigators observed that patients with higher circulating IL-6 levels had more severe RV dysfunction, despite a comparable severity of pulmonary vascular disease [56] (Table 2).

These observations suggest an inflammatory component to disease progression in RHD and PH, which might be developed as a response to chronic RHD-induced pressure overload and may contribute to aggravation and maintenance of RV and RA remodeling observed in RHD.

#### 3.3.2. Impact of Inflammation on Cellular and Molecular Remodeling in Patients with Cardiac Arrhythmias in RHD

Cardiac arrhythmias, particularly AF, are common complications of RHD and are strongly associated with adverse clinical outcomes [7]. Inflammation-induced fibrosis could be considered an important contributor to the arrhythmogenic substrate that predisposes patients with right heart remodeling to atrial arrhythmias, including AF [24,25]. Cellular remodeling affecting RA and RV cells, including CMs and FBs, constitutes an essential component of the arrhythmogenic substrate [4,46]. Although not focusing on RHD, an investigation involving isolated human cardiac FBs revealed that pro-inflammatory stimuli with TNF⍺ activated FB mRNA expression of IL-6, and IL-1β [57]. In the Multi-Ethnic Study of Atherosclerosis (MESA)-RV study involving 4009 patients, extreme elevations in the inflammatory biomarkers CRP and IL-6 were associated with an increased RV mass and volume [58] (Table 2). In the context of myocardial structural changes, such hypertrophy suggests a remodeling of the CMs in response to pressure–volume overload combined with the inflammatory stimuli [59].

It is important to note that data from patients with RHD and AF focusing on inflammation are limited, but the convergence of evidence and interpretation of basic data have helped to better understand the potential pathophysiological scenario that implicates the inflammatory status in the onset of AF.

#### 3.3.3. Inflammation-Triggered Electrical Abnormalities Responsible for Arrhythmias in Patients with RHD

In patients with elevated levels of IL-1, IL-6, and TNF-α, an investigation revealed that atrial expression of connexin (Cx) 40 and Cx43 was significantly decreased [60]. It has been suggested that perturbed expression and function of Cx40 and Cx43 might contribute to the arrhythmogenic substrate and increase AF vulnerability [61]. In patients with congenital heart disease, abnormal Cx40 and Cx43 expression was associated with perturbed conduction and vulnerability for cardiac arrhythmias [62]. In children with tetralogy of Fallot, disturbances in Cx43 expression and localization were suspected to influence heart embryogenesis and maturation, while contributing to the RV hypertrophy and dysfunction associated with arrhythmias [63]. It has been shown that patients with tetralogy of Fallot express elevated levels of inflammatory biomarkers, including CXCL6, that are associated with RV hypertrophy [64,65]. In addition, it has been proposed that inflammation might induce electrical dysfunctions characterized by the presence of fibrosis and low-voltage zones in the RV and RA in RHD [66,67] (Table 2). Hence, mounting evidence converges to highlight the potential contribution of inflammation in RHD-induced arrhythmogenic substrates.

**Table 2:** Clinical evidence of the implication of inflammation in arrhythmogenesis in RHD.

Clinical Study	Number of Patients Involved	Quantified Inflammatory Biomarkers	Associated Potential Arrhythmogenic Features	Ref.
Prospective study	29 patients with primary PH, 9 patients with PH secondary to COPD, 15 healthy controls	Increased serum levels of IL-1β, IL-6, TNF-α	Severe cardiopulmonary disease requiring pulmonary transplantation	[52]
Prospective study	112 COPD patients	Increased serum expression of hs-CRP, IL-6, and ET-1; decreased expression of IL-10	Not reported	[53]
Cross-sectional study	94 patients with COPD	Increased serum expression of IL-6 and hs-CRP	Low forced vital capacity, assessing poor cardiopulmonary health	[54]
Prospective study	172 patients with RHF	Increased plasmatic levels of soluble CD163, CXCL12	Severe RHF, increased RA pressure	[55]
Retrospective study	40 patients with PAH	Increased circulating levels of IL-6	RV malfunction, RA pressure overload	[56]
Multi-Ethnic Study of Atherosclerosis-RV prospective study	4009 patients studied for RV remodeling	Increased CRP and IL-6	Increased RV mass, RV end-diastolic volume, RV end-systolic volume, and RV stroke volume (RVSV)	[58]
Prospective study	32 patients with tetralogy of Fallot	Not reported, but discussed potential association with elevated levels of PDGF, TGFβ, IGF, FGF	Decreased level of Cx43, perturbed Cx43 localization, RV hypertrophy, and RV defect	[63]
Retrospective study	38 patients with tetralogy of Fallot	Increased expression of CXCL6, F3, SLC7A2, and SLC7A1	Reported myocarditis and RV malfunction	[64]
Prospective study	50 patients with tetralogy of Fallot	Increased levels of IGFBP7	RV malfunction and RV hypertrophy	[65]

Altogether, the molecular and electrophysiological changes associated with mechanical stress, oxidative injury, and inflammation might be responsible for alterations in the heart structure and function. Through their effects on fibrosis, endothelial integrity, and electrical signaling, pro-inflammatory cytokines might contribute to the progression of RHD and increase the risk of AF. These observations highlight the potential central role of inflammation in RHD and support the idea that targeting inflammatory cytokine activity could help to prevent or reduce atrial arrhythmias, including AF.

## 4. Implication of Anti-Inflammatory Cytokines in the Prevention of Arrhythmias and AF in RHD

### 4.1. Generalities

AF and RHD arise from complex electrophysiological and structural remodeling driven in part by an imbalance between pro- and anti-inflammatory mediators [7,9]. Pro-inflammatory cytokines promote electrical instability by altering ion channel expression, impairing Ca^2+^ handling, and facilitating atrial fibrosis through fibroblast activation [18].

In contrast, anti-inflammatory pathways, including cytokines like IL-10 and IL-37, as well as specialized pro-resolving mediators such as resolvin-D1 (RvD1) and maresin-1 (MaR1), act to counter these effects, with possible beneficial impacts in the heart [15,68,69,70].

Anti-inflammatory cytokines might play a fundamental role in balancing the inflammatory environment that drives right heart remodeling and AF [9]. Among the molecules naturally expressed in response to myocardial insults, some anti-inflammatory cytokines have emerged as key modulators capable of limiting myocardial injury, fibrosis, and arrhythmogenesis in both experimental and clinical studies [71].

In addition to anti-inflammatory cytokines, endogenous cytokine antagonists and soluble receptors play a central role in regulating the inflammatory balance in cardiovascular disease. Their potential impact in cardiovascular disease is discussed below.

### 4.2. Potential Role of Inflammation Resolution Promotor RvD1 in Preventing AF in RHD

Unlike classical anti-inflammatory drugs that suppress immune activation, RvD1 has been shown to actively promote the resolution phase of inflammation, restoring tissue homeostasis without compromising the host defense [72].

RvD1 is a bioactive autacoid derived from the omega-3 long-chain fatty acid DHA (eicosahexaenoic acid) [73]. RvD1 has been described to exert its beneficial effects through binding to the G-protein-coupled receptors ALX/FPR2 and GPR32, triggering downstream signaling that limits leukocyte infiltration, stimulates macrophage phagocytosis of apoptotic neutrophils, enhances macrophage-mediated efferocytosis, and reduces pro-inflammatory cytokine production [72,73]. In cardiovascular tissues, it has been demonstrated that RvD1 suppresses NF-kB activation, reduces NLRP3 activity, and decreases levels of IL-6, TNF-⍺, and IL-1β, thereby attenuating endothelial activation and oxidative stress [74].

**Figure 4:**
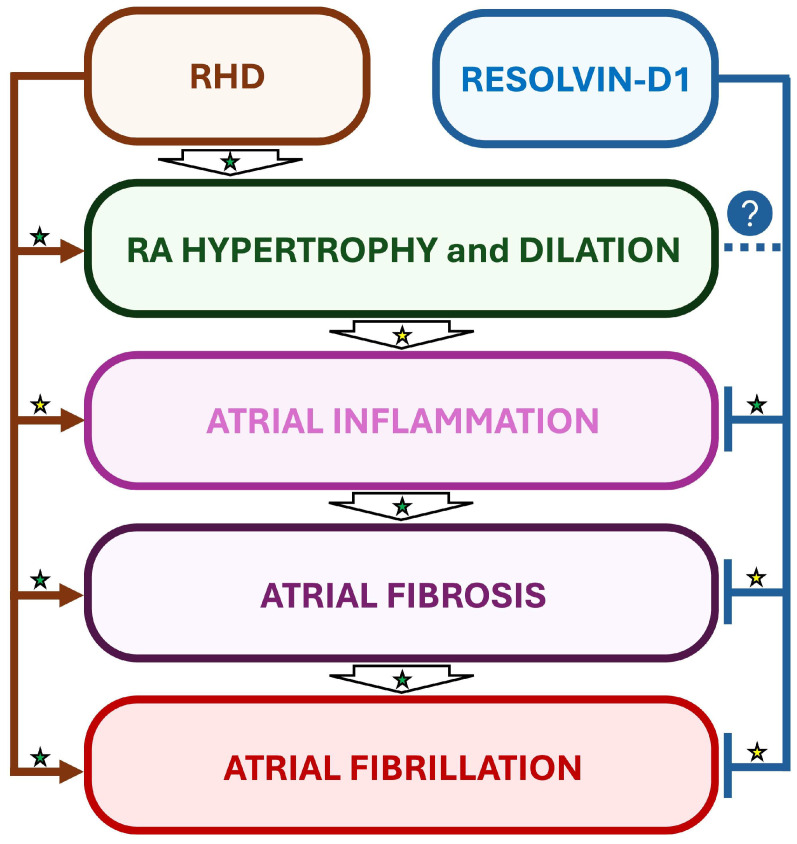
Suggested beneficial impact of RvD1-induced inflammation resolution in a rat model of RHD and AF. In a rat model of monocrotaline-induced right heart disease (RHD), resolvin-D1 (RvD1) has been demonstrated to prevent atrial inflammation and fibrosis, but not myocardial hypertrophy. Interestingly, RvD1’s effects on inflammation and fibrosis were accompanied with attenuation of AF vulnerability in RHD. Established mechanisms in human pathophysiology are identified by a green star. Emerging/speculative pathways based on preclinical evidence are identified by a yellow star. Question mark with blue circle indicates a preventive effect of RvD1 which requires further demonstration.

Interestingly, in a rat model of RHD and AF induced by monocrotaline (MCT), daily treatments with RvD1 starting one day before MCT administration did not prevent MCT-induced RV and RA hypertrophy, but attenuated the atrial inflammation and significantly decreased AF inducibility, suggesting the importance of early treatment of inflammation for optimized beneficial effects [15] (Figure 4).

In contrast, some clinical studies involving omega-3 administration in patients at risk of cardiovascular disease have reported contradictory results. In the Reduction of Cardiovascular Events with Icosapent Ethyl Intervention Trial (REDUCE-IT), high-dose omega-3 fatty acid supplementation with highly purified icosapent ethyl (4 g) was associated with decreased risk of cardiovascular events, but no benefits on AF [75]. The Statin Residual Risk with Epanova in High-Cardiovascular-Risk Patients with Hypertriglyceridemia (STRENGTH) trial, which used a combination of EPA (eicosapentaenoic acid) and DHA, showed no significant effect of omega-3 fatty acids on the composite CV endpoint, nor beneficial effects on AF inducibility [76].

Interestingly, none of the studies involving DHA or EPA have reported whether the methodology implicated a strategy promoting the metabolism of these fatty acids into their active metabolites, respectively the D-series and E-series resolvins. In addition, quantifications of these active forms responsible for the effective resolution of inflammation were not provided in trials involving DHA or EPA.

More investigations are required to assess the impact of therapeutic strategies involving RvD1 in the management of inflammation and AF in RHD.

### 4.3. Endogenous Antagonists of IL-1 Receptor

The IL-1 receptor antagonist (IL-1Ra) represents a major natural inhibitor of IL-1 signaling by competitively blocking IL-1α and IL-1β binding to IL-1 receptor type I (IL-1R1), thereby limiting downstream inflammatory and fibrotic responses [77,78,79].

In a murine model of IL-1 receptor (IL1-R1) deficiency, a myocardial infarction provoked by ligation of the left anterior descending coronary artery (LAD) was associated with less LV malfunction, decreased CM apoptosis, and limited LV enlargement, confirming an important contribution of inflammatory signaling in ischemic and fibrotic cardiac disease [80]. Interestingly, LAD in IL-1Ra-deficient animals provoked a larger LV myocardial infarction size and aggravated cardiac disease associated with LV enlargement, demonstrating the importance of this endogenous IL-1 receptor antagonist in attenuating inflammation and inflammation-associated cardiac remodeling [80]. In a murine model of Kawasaki disease, IL-1Ra contributed to preventing myocardial dysfunction and ventricular arrhythmia [81].

In 88 epicardial adipose tissue samples obtained from patients with coronary syndrome, increased levels of IL-1β were associated with coronary artery disease [79]. Interestingly, acute coronary syndrome was associated with a loss of the counter-regulatory activity of IL-1Ra, in contrast with the pro-inflammatory effects of IL-1β activation [79]. In term of the prognosis, quantification of plasmatic levels of IL-1Ra in 323 patients revealed that high levels of IL-1Ra were independently associated with an increased mortality in patients with a myocardial infarction after 2 and 5 years [82].

These observations highlight the importance of further investigations to better understand the impact of anti-inflammatory strategies involving IL-1Ra in RHD and AF.

### 4.4. Endogenous Modulation of TNF-α Receptor

Soluble tumor necrosis factor receptors (sTNFR1 and sTNFR2) modulate TNF-α bioavailability and signaling by acting as decoy receptors [83,84]. In a prospective cohort of 251 patients with an ST-segment elevation myocardial infarction, circulating sTNFR levels were associated with disease severity and ventricular malfunction, highlighting their role as both biomarkers and regulators of TNF-α-mediated myocardial inflammation [85]. In explanted hearts obtained from patients with dilated cardiomyopathy, congestive heart disease, or ischemic heart disease, TNF receptor proteins were shown to be dynamically regulated, suggesting that failing hearts express elevated levels of TNF-α, and that endogenous lower expression of this pro-inflammatory agent may promote beneficial effects [86].

Although their specific involvement in right heart remodeling and atrial arrhythmogenesis remains insufficiently characterized, these endogenous modulators are essential components of the pro-/anti-inflammatory balance shaping cardiac remodeling [87].

### 4.5. Involvement of IL-10 in Potentially Preventing Cardiac Remodeling and Arrhythmogenesis

**Figure 5:**
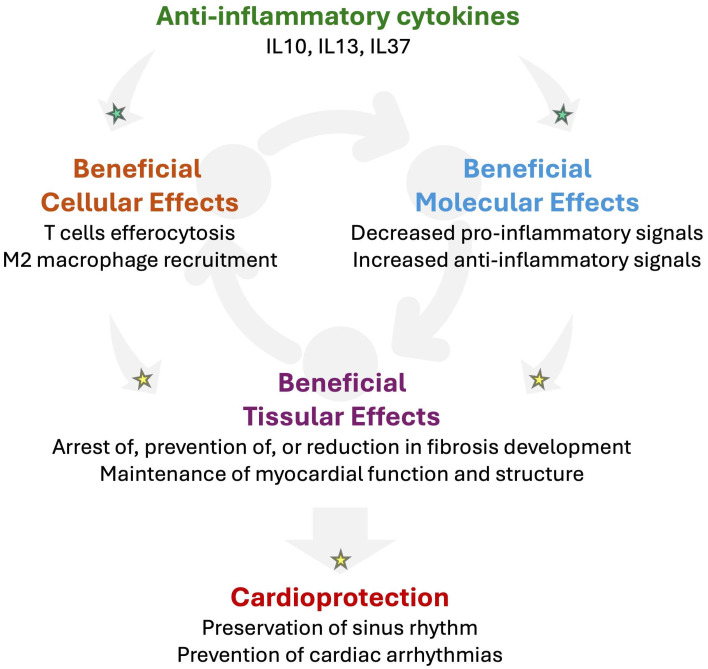
Potential beneficial impact of anti-inflammatory cytokines in RHD to prevent arrhythmogenesis. Mounting evidence suggests that anti-inflammatory cytokines such as interleukin-(IL)-10, IL13, or IL37 might promote cardio-protection by promoting T cell-impaired efferocytosis and M2 macrophage recruitment in injured tissue. In addition, anti-inflammatory cytokines may activate the secretion of further anti-inflammatory cytokines while decreasing pro-inflammatory signals. Such an anti-inflammatory response might contribute to reducing fibrosis and preserving the myocardial function and structure. Altogether, anti-inflammatory signaling has the potential to prevent the development of the arrhythmogenic substrate, which reduces the vulnerability for cardiac arrhythmias. Established mechanisms in human pathophysiology are identified by a green star. Emerging/speculative pathways based on recent preclinical and clinical evidence are identified by a yellow star.

IL-10 is a potent immunoregulatory cytokine produced by macrophages, regulatory T cells, and other immune cells in response to inflammatory stimuli; it functions to limit inflammation and promote immune homeostasis [88]. IL-10 interacts with the heterotetrameric complexes of the IL-10 receptors IL-10Rα (two subunits) and IL-10Rβ (two subunits), which induce STAT3 signaling to promote anti-inflammatory effects [89]. In cardiac surgery, studies have examined cytokine responses, including IL-10, but the current evidence does not consistently show that higher plasma IL-10 is associated with a lower incidence of postoperative AF or improved ventricular recovery [90]. Clinical studies in pulmonary arterial hypertension (PAH) subtypes, including connective tissue disease-associated PAH, have reported changes in the circulating IL-10 levels compared with healthy controls, reflecting immune dysregulation in PAH [91]. Experimental data in myocardial injury models show that IL-10 can attenuate inflammation, reduce fibrosis, and improve ventricular remodeling and function, whereas reduced IL-10 activity is associated with increased inflammation and worse outcomes in these settings [92] (Figure 5).

A systematic review of 286 patients with IL-10 and/or IL-10 receptor deficiencies revealed that absence of IL-10 was associated with chronic auto-inflammation and failure to thrive [93]. In a study involving 218 patients with osteoarthritis, the serum levels of IL-10 were significantly low [94].

Although experimental studies have reported a potential beneficial impact of IL-10 in preventing AF vulnerability [68], clinical data reporting any positive role of therapies targeting IL-10 are lacking, specifically in the context of AF and RHD. Such a poorly explored area suggests an interesting avenue for future investigations.

### 4.6. Impact of IL-13 in Reducing Cardiac Remodeling Associated with Heart Failure

IL-13 is a T helper 2 (Th2) cytokine with anti-inflammatory properties [95]. In a murine model of myocardial injury such as viral myocarditis, IL-13 has been shown to reduce cardiac damage and improve ventricular function in part, by enhancing M2 macrophage polarization [96]. Experimental evidence suggests that mast cells can produce IL-13 and other mediators that modulate macrophage and FB function in cardiac injury models, but definitive translational studies showing that IL-13 secretion by cardiac mast cells limits extracellular matrix deposition and preserves conduction in pressure overloads are not well established [96,97]. In a murine model of IL13 deficiency, it has been demonstrated that administration of recombinant IL-13 is involved in amelioration of neonatal cardiomyocyte cell cycle activity and promotion of heart regeneration [98].

In a clinical study involving 93 patients with advanced heart failure supported with a left ventricular assist device (LVAD), lower levels of inflammation marked by reduced circulating levels of TNFα, IL-5, IL-6, IL-7, and interferon gamma (IFNγ) were accompanied by a lower anti-inflammatory response, identified by low circulating levels of IL-13, associated with subsequent structural and functional cardiac recovery following mechanical unloading [99]. In patients with ischemic heart disease, the levels of IL-13 were significantly lower than those of healthy controls [100]. Interestingly, the same study reported that patients with ischemic heart disease had high serum levels of pro-inflammatory biomarker IL18 [100]. In contrast, in a cohort of 3704 patients, two haplotypes in *IL13* (ATG and ATA) were associated with an increased risk of coronary artery disease compared to healthy patients [101] (Figure 5).

Although limited data exist about the role of IL-13 in RHD and AF, its potential impact in other cardiovascular diseases as reported above suggests a potential area of investigation for the development of future anti-inflammatory approaches.

### 4.7. Effect of IL-37 in Preventing Cardiac Remodeling and Arrhythmias

IL-37, a member of the IL-1 cytokine family, is a potent suppressor of innate inflammatory pathways, especially IL-18-dependent signaling, and it is being increasingly implicated as a systemic anti-inflammatory mediator with potential cardiovascular relevance [102]. Experimental studies have demonstrated that IL-37 suppresses IL-6 and TNF-α signaling and promotes immune-regulatory responses, including enhanced Treg-associated pathways, leading to attenuation of inflammatory cardiac hypertrophy in translational models [103,104,105]. In a murine model of diabetic cardiomyopathy, IL-37 was shown to ameliorate myocardial fibrosis by regulating mtDNA-enriched vesicle release [106]. In addition, IL-37 was shown to prevent isoproterenol-induced cardiac remodeling by suppressing myocardial inflammation and oxidative stress via modulation of JAK2/STAT3 signaling, in a murine model of cardiac hypertrophy [107]. However, the direct effects on the atrial arrhythmogenic substrate remain under investigation.

In a cohort of 167 participants, it was shown that AF patients exhibited significantly elevated circulating and peripheral blood mononuclear cell (PBMC) levels of IL-37, particularly in paroxysmal and persistent AF, which correlated with increased levels of systemic inflammatory markers and disease burden, suggesting a compensatory anti-inflammatory reaction as a defense response during arrhythmogenesis [108]. Experiments performed ex vivo in the same study demonstrated that recombinant IL-37 suppresses IL-6 production and systemic inflammatory responses in cells from AF patients, supporting its anti-inflammatory activity, but direct cardio-protective effects within atrial tissue remain to be established [108] (Figure 5).

Although these studies suggest a beneficial impact of IL37 against cardiac hypertrophy, myocardial inflammation, and fibrosis, its impact in the specific context of RHD and AF remains under investigation.

## 5. Integration of Inflammation-Targeted Strategies into Contemporary Atrial Fibrillation Management in the Context of RHD

### 5.1. Prerequisites

Management of AF in the setting of RHD remains challenging, owing to advanced atrial remodeling, limited efficacy of rhythm control strategies, and frequent intolerance of arrhythmia recurrence [7]. Current guideline-directed AF management primarily focuses on symptom control, stroke prevention, and modification of traditional risk factors [7]. However, as described above, growing evidence suggests that inflammation represents an important, albeit heterogeneous, contributor to atrial remodeling and AF vulnerability in RHD. This raises the question of how inflammation-targeted approaches could be realistically incorporated into contemporary AF management paradigms.

### 5.2. Diagnostic Integration: Inflammatory Phenotyping in RHD-Associated AF

In RHD, inflammatory activation is closely intertwined with an increased pressure and volume overload, oxidative stress, and chronic dilation of the right myocardial structure [6]. Rather than acting as an isolated causal driver, inflammation appears to function as a disease modifier that amplifies atrial structural and electrical remodeling [25,28]. Accordingly, inflammation-targeted strategies should be viewed as adjunctive, phenotype-guided interventions integrated within existing diagnostic and therapeutic algorithms, rather than as stand-alone treatments for AF.

Incorporation of inflammation into AF management might begin with improved identification and phenotyping of the inflammatory status. Circulating inflammatory biomarkers—such as hs-CRP, IL-6, TNF-α, and composite indices (e.g., the neutrophil-to-lymphocyte ratio)—are frequently elevated in RHD and correlate with the disease severity and adverse outcomes such as atrial arrhythmias [15,25,28]. While these inflammatory markers lack specificity, their integration with the clinical context and imaging may help identify patients with an “inflammatory-active” atrial substrate.

Advanced cardiac imaging plays a central role in this approach. A cardiac magnetic resonance assessment of the RA size, function, and low-voltage zones eventually due to fibrosis provides critical insight into the stage of atrial cardiomyopathy and conduction properties [109]. Patients with evidence of active remodeling and limited fixed fibrosis may represent a subgroup in whom transient modulation of inflammation could meaningfully influence AF susceptibility or progression. Conversely, extensive fibrosis likely reflects an irreversible substrate in which inflammation-targeted strategies are unlikely to confer substantial benefits [110].

### 5.3. Therapeutic Integration of Anti-Inflammatory Approaches Across Current AF Management Stages: Importance of the Timing in Accordance with RHD’s Stage of Aggravation

#### 5.3.1. Upstream and Preventive Strategies

In early RHD or paroxysmal AF, upstream interventions could aim to reduce the inflammatory burden and complement current strategies of hemodynamic optimization [15]. Effective treatment of PAH, relief of a right-sided volume or pressure overload, correction of hypoxemia, and management of systemic congestion may contribute to indirectly attenuating inflammatory signaling, activated in direct response to structural remodeling, and slow atrial electrical changes [111]. Lifestyle and comorbidity management—although understudied in RHD—may also contribute to reducing chronic inflammatory activation, which might contribute to preventing cardiac arrhythmogenesis [7].

#### 5.3.2. Rhythm Control and Catheter Ablation

Catheter ablation in RHD is associated with lower success rates and higher recurrence compared with left-heart-predominant AF, largely due to advanced RA remodeling [112]. Due to the local insult generated by the catheter ablation procedure per se, inflammation may further compromise the ablation efficacy by promoting early recurrence, lesion instability, and ongoing substrate evolution [113]. In this context, short-term, peri-procedural anti-inflammatory strategies could theoretically reduce early arrhythmia recurrence and improve procedural durability, particularly in patients with evidence of active inflammatory remodeling [114]. Importantly, such approaches should be carefully timed to consider the dynamics and kinetics of the inflammatory molecules and cells that might be targeted for AF prevention.

#### 5.3.3. Rate Control and Advanced RHD

In advanced RHD with persistent AF and extensive atrial fibrosis, current inflammation-targeted strategies might not be sufficient to reverse an established arrhythmogenic substrate [15,25]. In RHD patients, relevant management could prioritize symptom relief, prevention of tachycardia-induced cardiomyopathy, and optimization of RV function [115]. In this context, future anti-fibrosis strategies aiming to restore RA and RV function may be efficient as synergic medication used as a complementary approach to the current rate control therapies [115,116].

## 6. Discussion

### 6.1. Complexity of Inflammation in AF and RHD

AF is being increasingly recognized as an inflammatory-driven arrhythmia in which innate immune activation may play a central role in promoting atrial structural and electrical remodeling [117]. Among inflammatory signaling, IL-1-family cytokines such as IL-1β were shown to promote arrhythmias [118], particularly IL-18, which functions as a key pro-inflammatory mediator that amplifies macrophage activation, fibrotic signaling, and arrhythmogenic substrate formation [119,120,121]. In parallel, several endogenous counter-regulatory cytokines—including IL-37, IL-10, and IL-13—are up-regulated under inflammatory cardiovascular conditions and appear to function as compensatory brakes on excessive immune activation [103,122,123]. IL-37, a member of the IL-1 family, broadly suppresses innate immune signaling through inhibition of IL-18-dependent pathways and downstream cytokines such as IL-6 and TNF-α, and is elevated in patients with AF in proportion to the inflammatory burden [122]. IL-10, produced by regulatory T cells and anti-inflammatory macrophages, contributes to immune resolution and attenuation of inflammatory cardiac remodeling, while IL-13 modulates macrophage polarization and inflammatory responses in a context-dependent manner [123]. Collectively, the available experimental and clinical evidence suggests that increased expression of these anti-inflammatory cytokines in AF reflects an insufficient compensatory response to sustained IL-18-driven inflammation, rather than a uniformly protective mechanism [124].

In contrast, some inflammatory biomarkers such as IL2 and IL4 have shown contradictory effects, revealing pro-inflammatory action under some conditions while suggesting anti-inflammatory benefits in other studies [125,126,127,128]. In high-risk acute coronary syndrome, immunomodulation with low-dose IL2 was shown to produce a significant reduction in arterial inflammation compared to a placebo [128,129]. Larger trials are needed to confirm IL2’s impact on the CV outcomes. In a mouse model of myocardial infarctions, IL4 was shown to attenuate cardiac hypertrophy and enhance myocardial repair [130].

RHD can result from various risk factors, including PH, left-heart-related RHD, and primary pulmonary vascular diseases, which differ markedly in their inflammatory profiles and arrhythmogenic mechanisms [3]. In PH, a chronic RV pressure overload is tightly coupled to a pulmonary and cardiac pro-inflammatory milieu characterized by activation of innate immune pathways, endothelial dysfunction, and elevated cytokines such as IL-6, IL-1β, and TNF-α, which might promote myocardial fibrosis, microvascular rarefaction, and ion-channel remodeling, thereby creating a substrate for atrial and ventricular arrhythmias [38].

In left-heart-related RHD, post-capillary PH and venous congestion lead to RA stretch and neurohormonal activation, where systemic inflammation—often driven by comorbid heart failure and metabolic disease—might act as a modulator of structural and electrical remodeling rather than a primary driver [131].

Conversely, primary pulmonary vascular diseases, such as idiopathic PAH, display distinct immune signatures with autoimmune features, perivascular inflammation, and dysregulated adaptive immunity, which contribute not only to pulmonary vascular remodeling, but also to myocardial inflammation and fibrosis, potentially explaining early arrhythmic vulnerability [132].

Beyond PAH, genetic cardiomyopathies such as congenital heart disease, tetralogy of Fallot, and channelopathies affecting the right heart, such as arrhythmogenic right ventricular cardiomyopathy, can exhibit inflammation secondary to desmosomal dysfunction, immune activation, and myocyte loss, which further amplifies their electrical instability [133].

Together, these data highlight that arrhythmogenesis in RHD is highly context-dependent, arising from the interaction between disease-specific inflammatory pathways and mechanical stress, underscoring the need for etiology-tailored anti-inflammatory and anti-arrhythmic strategies.

### 6.2. Paradox of Right-Heart-Specific Anti-Inflammatory Approaches: State of Evidence and Limits

Very few data about the implication of IL2 and IL4 in AF or RHD are available, suggesting an important need for further investigations on this topic.

Various clinical trials have explored anti-inflammatory medications in cardiac diseases associated with AF, with mitigated results. In a clinical trial involving 241 patients undergoing coronary artery bypass graft surgery and aortic valve replacement, administration of 100 mg hydrocortisone intravenously for 3 days was associated with reduced incidence of AF post-surgery [134]. In the Colchicine for the Prevention of the Post-Pericardiotomy Syndrome (COPPS) study involving 336 patients, 0.5 mg of colchicine twice daily was associated with reduced occurrence of postoperative AF after cardiac surgery [135]. In contrast, administration of 0.6 mg colchicine twice daily did not prevent AF recurrence post-catheter ablation in a randomized placebo-controlled trial involving 199 patients [136]. Interestingly, the DECLARE-TIMI 58 (Dapagliflozin Effect on Cardiovascular Events—Thrombolysis in Myocardial Infarction 58) trial involving 17,160 patients revealed SGLT2 inhibitor dapagliflozin AF in patients with atherosclerosis, cardiovascular disease, and heart failure [137].

These data were advocated by evidence obtained in fundamental research, where high-dose dapagliflozin prevented Cx43 remodeling and vulnerability for ventricular arrhythmias in a rat model of MCT [138]. Recently, our group described that RHD is accompanied by progressive myocardial inflammation and that early treatment with RvD1 could attenuate inflammation and reduce AF vulnerability. These data suggest that targeting the pro-inflammatory versus anti-inflammatory imbalance in RHD may represent a novel strategy to limit atrial remodeling and disease progression [15]. Studies have suggested that specialized pro-resolution mediators such as RvD1 and their omega-3 metabolic precursor might have a beneficial impact on modulating hypercontractility, even in airway diseases [139]. Nevertheless, in the REDUCE-IT study, icosapent ethyl, a highly purified eicosapentaenoic acid ethyl ester, decreased the risk of ischemic events, including cardiovascular death, but increased the incidence of atrial tachyarrhythmias, including AF (3%), versus a control (2%) [140]. Although this trial was not performed in RHD patients, the results obtained contrasted with evidence observed in animal models of RV and RA remodeling.

RHD is a multifactorial condition, and despite the mounting evidence of an implication of inflammation, few (if not no) clinical trials have studied anti-inflammatory medications in RHD and AF. This field is growing, and promising results have been obtained in preclinical investigations, suggesting that further research is required to consolidate the understanding of the impact of anti-inflammatory cytokines and pro-resolution strategies in RHD and arrhythmias, including AF.

### 6.3. Optimization of Intervention Timing and Patient Stratification

Despite a strong mechanistic rationale, clinical trials targeting inflammation in AF have yielded mixed or neutral results, underscoring the complexity of inflammatory signaling and the risks of nonspecific immunomodulation [141]. Patients with RHD may be particularly vulnerable to adverse effects related to systemic anti-inflammatory therapies, including an infection risk, hepatic dysfunction, and a fluid imbalance [142]. Therefore, broad cytokine inhibition cannot currently be recommended to manage arrhythmogenicity in RHD. Future strategies will likely require precision approaches, targeting specific inflammatory pathways, defined patient subsets, and limited therapeutic windows. Integration of biomarker-guided selection and imaging-based substrate characterization will be essential to avoid adverse effects and to maximize the potential benefit.

Prospective studies are needed to define inflammatory AF phenotypes specific to RHD, establish causal links in human atrial tissue, and determine whether targeted modulation of inflammation can meaningfully alter the AF trajectory or improve the clinical outcomes. Importantly, such strategies should be tested as adjuncts to, rather than replacements for, established guideline-directed current anti-arrhythmic therapies.

## 7. Limitations

Despite growing recognition of the specificities of the right heart’s role in cardiovascular disease, clinical data specifically addressing AF in the context of RHF remain remarkably limited. Most AF studies have focused on left-sided heart disease, with RHF populations either underrepresented or analyzed only as secondary subgroups. Consequently, the true prevalence, natural history, and prognostic impact of AF in RHD and RHF, particularly in conditions such as PH, congenital heart disease, and isolated RV dysfunction, are poorly defined.

Moreover, therapeutic strategies for rhythm and rate control in RHD and RHF are largely extrapolated from left heart failure paradigms, despite fundamental differences in atrial anatomy, atrial remodeling, cardiac chamber hemodynamics, and atria-specific arrhythmogenic mechanisms.

This lack of RHD-specific clinical evidence represents a critical knowledge gap that hinders personalized management and underscores the need for dedicated observational studies and interventional trials focusing on AF in RHD.

In terms of inflammation, human data directly linking specific cytokines to AF in RHD remain limited and are largely observational. Mechanistic interpretations should therefore be viewed as potential future research avenues. This narrative review mainly aims to propose original directions that could be explored in subsequent investigations.

## 8. Conclusions

Cardiac inflammation and fibrosis have been observed among atrial and ventricular remodeling associated with arrhythmogenesis in RHD. Although mounting evidence supports an important role of inflammation in cardiac arrhythmias, including AF, new strategies are required to identify the specific inflammatory signals and pathways involved in the arrhythmogenic substrate in RHD. Overall, inflammation represents a key modifier of the electrical conduction and RA dilation associated with AF vulnerability. Integration of inflammation-targeted strategies into contemporary AF management should be selective, adjunctive, and guided by careful phenotyping in a personalized medicine manner. While the current evidence does not support routine clinical use, a precision-based approach combining inflammatory biomarkers, advanced imaging, and optimized hemodynamic management holds promise for improving AF care in this specific RHD population.

## 9. Take-Home Message and Future Directions

- Inflammation is an under-recognized contributor to AF in RHD, promoting RA fibrosis and electrical instability.
- Inflammatory biomarkers correlate with the RHD severity and potentially the AF burden, supporting their use for risk stratification and prevention.
- Integrating inflammation-targeted strategies may improve AF management in RHD in combination with current anti-arrhythmic strategies.

## Figures and Tables

**Table 1:** Selected animal models of RHD associated with right-myocardial inflammation.

Animal Species	RHD Model	Identified Inflammatory Biomarkers	Associated Arrhythmogenic Features	References
Rat	Monocrotaline	Increased RA or/and RV expression of NLRP3 inflammasome, IL1β, CXCL1, TGFβ3, and TNF-α	RA and RV fibrosis, myocardial conduction slowing, a prolonged action potential duration, increased AF vulnerability	[15,25,29]
Rat	Sugen hypoxia	Increased RV expression of TNF-⍺, CRAR1, α-SMA, and CCN2	RV hypertrophy, RV fibrosis	[25]
Dog	Pulmonary artery banding	Increased RV expression of IL-1β and IL-6 Decreased expression of IL-10 and IL33	RV failure Increased oxidative stress and hypoxia (decreased heme-oxygenase 1)	[28]
Mouse	Thrombin	Increased PA expression of plasminogen activator inhibitor-1	Pulmonary embolism, increased RV systolic pressure	[31]

## Data Availability

Not applicable.

## Abbreviations

The main abbreviations used in this manuscript are as follows:

AF	Atrial fibrillation
Ca^2+^	Calcium
CMs	Cardiomyocytes
ECs	Endothelial cells
ECM	Extracellular matrix
FBs	Fibroblasts
RA	Right atrium
RHD	Right heart disease
RvD1	Resolvin-D1
TNF⍺	Tumor necrosis factor alpha
